# Harnessing lactic acid bacteria for nicotinamide mononucleotide biosynthesis: a review of strategies and future directions

**DOI:** 10.3389/fmicb.2024.1492179

**Published:** 2024-12-13

**Authors:** Linghui Kong, Xinyu Li, Taiyu Liu, Qingshou Yao, Jiayang Qin

**Affiliations:** ^1^School of Pharmacy, Binzhou Medical University, Yantai, China; ^2^Lab of Biorefinery, Shanghai Advanced Research Institute, Chinese Academy of Sciences, Shanghai, China

**Keywords:** lactic acid bacteria, nicotinamide mononucleotide, biosynthesis, metabolic engineering, microbial fermentation

## Abstract

Nicotinamide mononucleotide (NMN), one of the crucial precursors of nicotinamide adenine dinucleotide, has garnered considerable interest for its pharmacological and anti-aging effects, conferring potential health and economic benefits for humans. Lactic acid bacteria (LAB) are one of the most important probiotics, which is commonly used in the dairy industry. Due to its probiotic properties, it presents an attractive platform for food-grade NMN production. LAB have also been extensively utilized to enhance the functional properties of pharmaceuticals and cosmetics, making them promising candidates for large-scale up synthesis of NMN. This review provides an in-depth analysis of various metabolic engineering strategies, including enzyme optimization, pathway rewiring, and fermentation process enhancements, to increase NMN yields in LAB. It explores both CRISPR/Cas9 and traditional methods to manipulate key biosynthetic pathways. In particular, this study discussed future research directions, emphasizing the application of synthetic biology, systems biology, and AI-driven optimization to further enhance NMN production. It provides invaluable insights into developing scalable and industrially relevant processes for NMN production to meet the growing market demand.

## 1 Introduction

With the rapid development of modern medicine, the mean life expectancy of human beings has risen sharply (United Nations, 2019; Morrison and Malik, 2023). According to the World Health Organization, it is expected that by 2050, the global population over 65 years of age will reach 150 million. Nevertheless, with the increase in the elderly population, the incidence of age-related ailments such as Alzheimer’s disease, Parkinson’s disease, diabetes, cancer, and others is increasing, which could impose a substantial strain on healthcare systems. Consequently, the global market value and demand for anti-aging nutraceuticals have risen dramatically.

### 1.1 What is nicotinamide mononucleotide?

**Figure 1:**
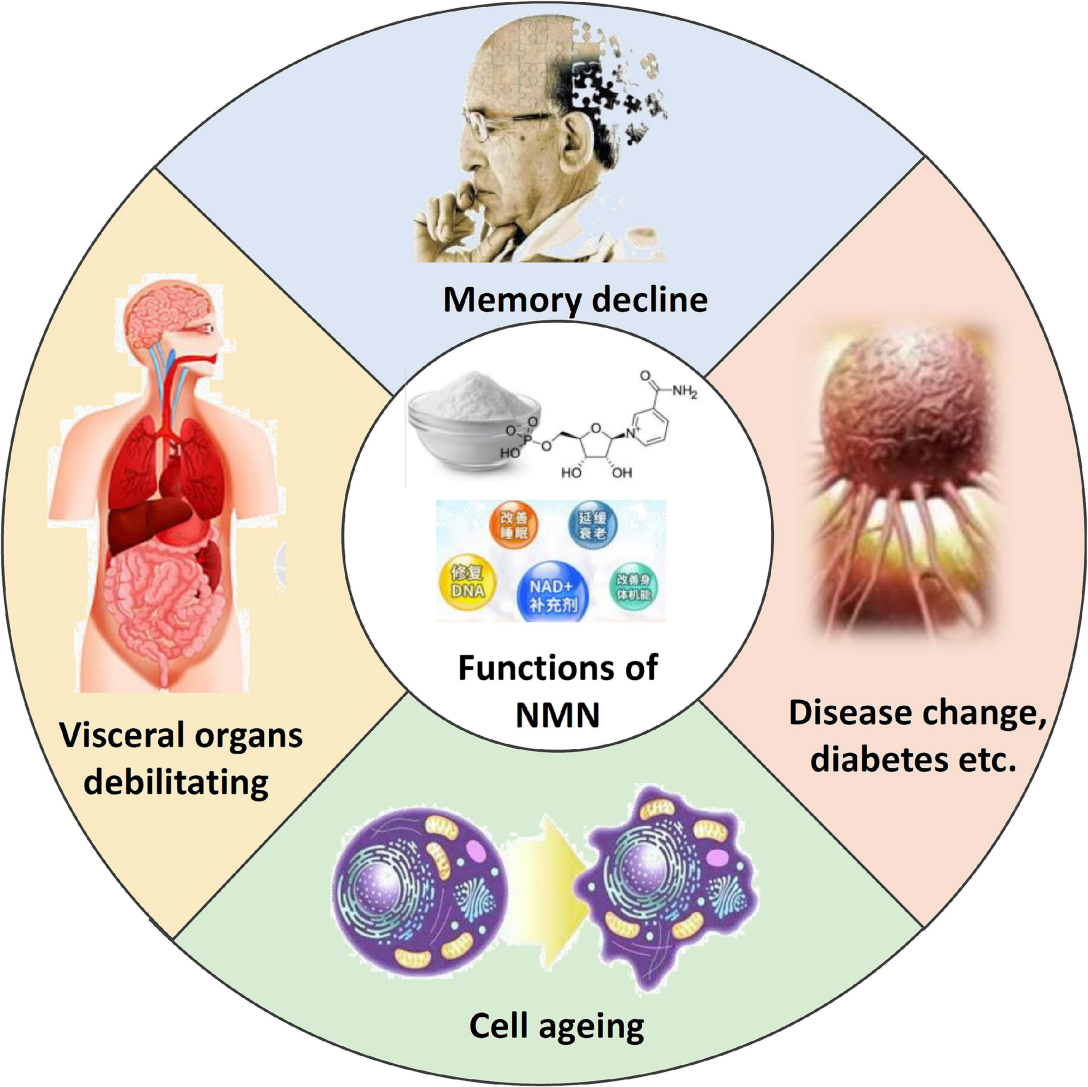
Schematic representation of the functions of NMN. Illustrates the key physiological roles of NMN in cellular processes.

Nicotinamide mononucleotide (NMN) is a biologically active nucleotide that plays an important role in cellular energy production (Sun et al., 2024; Yamaguchi et al., 2019). It is an essential precursor for the biosynthesis of nicotinamide adenine dinucleotide (NAD), a pivotal metabolic redox coenzyme that has garnered significant attention in recent years due to its biological value (Figure 1) (Nadeeshani et al., 2022). The concentration of NAD declines with age, affecting longevity and age-related health in mammals through energy metabolism as well as the activation of poly(ADP-ribose) polymerase and sirtuin protein (Jasper, 2013; Liu et al., 2018; Musi, 2023). The dietary supplement NMN can increase the level of NAD and reduce the incidence of age-related diseases such as Alzheimer’s disease and cardiovascular disease (Jablonska et al., 2022; Yao et al., 2017; Yi et al., 2023; Zhang J, et al., 2024). Therefore, the global NMN market demand continues to grow at a high speed, and NMN has attracted much attention as a promising anti-aging healthcare product (Wang et al., 2014). It is expected that by the end of the year, it will rise to 27.013 billion yuan with a growth rate of nearly 70.25%, so the efficient synthesis of NMN is very important for its industrial application (Liu, 2022).

### 1.2 Comparison of NMN synthetic methods

**Figure 2:**
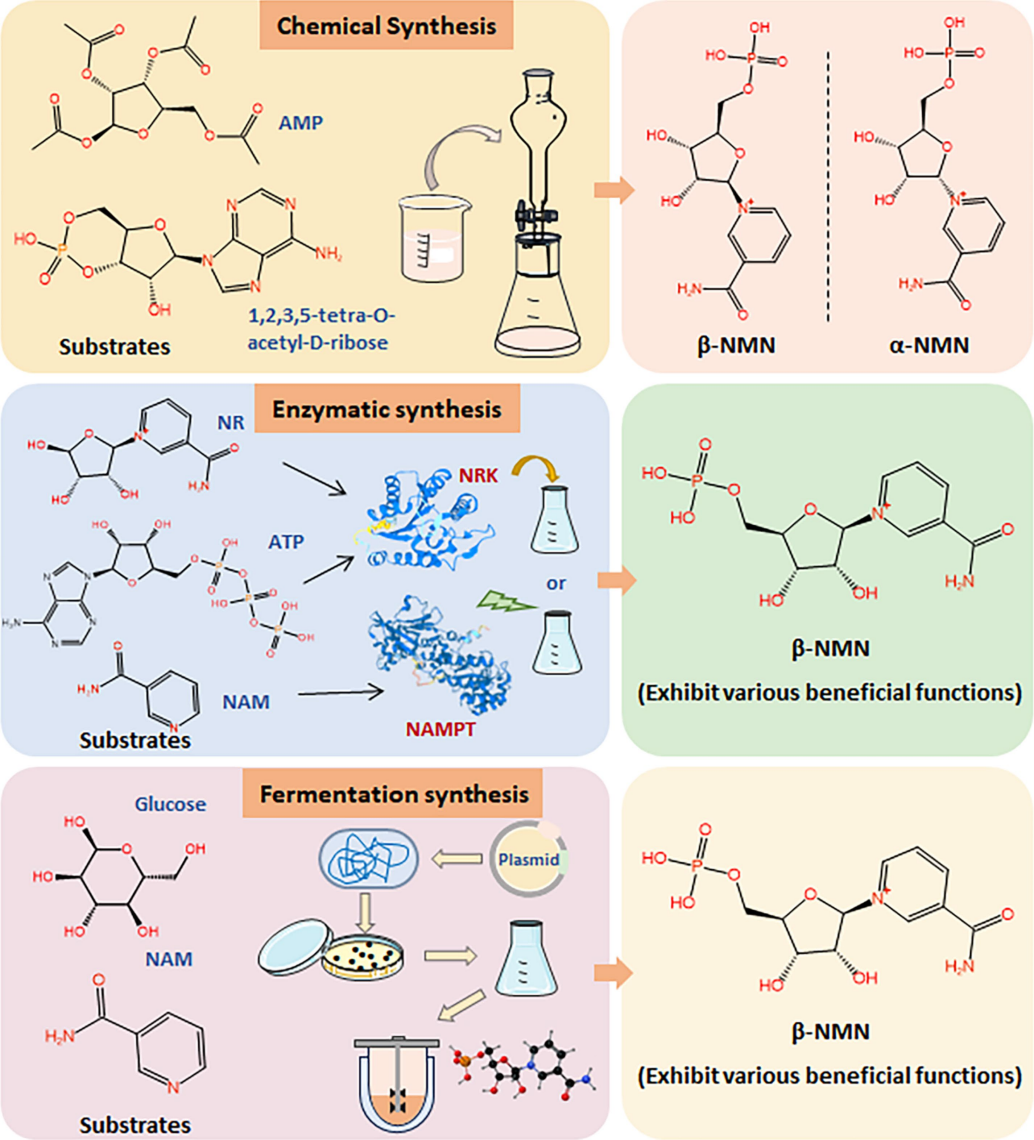
Synthesis methods of NMN. Comparison of chemical synthesis, enzymatic synthesis, and fermentation synthesis of NMN using different substrates.

As interest in NMN for its health benefits continues to surge, driven by its role in promoting longevity and cellular health, there is a critical need for cost-effective, scalable, and safe production methods. Chemically synthesized NMN, especially when intended for human consumption or medical applications, is subject to stringent regulatory scrutiny on safety, purity, and environmental impact. It can slow the pace of market approval, underscoring the need for biosynthetic NMN (Figure 2). The development of biosynthetic methods for NMN synthesis is the key issue for its industrial application in the food field (Kong et al., 2023; Liu et al., 2023; Luo et al., 2023). Various biological approaches have been developed for the efficient production of NMN, primarily focusing on enzymatic and microbial fermentation (Figure 2) (He et al., 2022; Huang et al., 2022; Zhou et al., 2022). NMN is synthesized through the riboside kinase (NRK)-mediated conversion of nicotinamide ribose (NR) and ATP (Cheng et al., 2024). Additionally, nicotinamide phosphoribosyltransferase (NAMPT) catalyzes the conversion of 5′-phosphate ribosyl-phosphoribosyl-1′-pyrophosphate (PRPP) and nicotinamide (NAM) to generate NMN (Fu and Zhang, 2019; Gardell et al., 2019; Zhu et al., 2022). However, the purification and stability of enzymes may face complex and costly issues in industrial production.

### 1.3 Advantages and challenges of NMN biosynthesis by lactic acid bacteria

Recent studies have identified the presence of the NMN metabolic pathway in *Escherichia coli*, enabling significant enhancements in NMN production through synthetic biology and metabolic engineering strategies (Bi et al., 2023). Nevertheless, the potential for *E. coli* to produce enterotoxins poses risks to human health, limiting its suitability for food-grade applications. In contrast, lactic acid bacteria (LAB) are acknowledged as safe probiotics, which can colonize the intestinal tract to improve intestinal health, and have a high application value in important fields closely related to human life such as agriculture, food, and medicine. Probiotic LAB, serving as an edible and safe microbial chassis cell, exhibit the potential for producing high-value compounds such as conjugated linoleic acid and exopolysaccharides (Kong et al., 2021; Xiong et al., 2019; Xiong et al., 2018). LAB are particularly well suited for NMN production due to their genetic tractability and capacity to efficiently convert inexpensive substrates, such as fructose and glucose. Several LAB species, including *Lactococcus lactis*, *Streptococcus thermophilus*, and *Lactiplantibacillus plantarum*, have successfully implemented CRISPR/Cas9 systems for precise genome editing (Kong et al., 2022a; Kong et al., 2022b; Xiong et al., 2020). The genus *Fructobacillus* can utilize fructose in its metabolic process, thereby enhancing the efficiency of NMN biosynthesis (Sugiyama et al., 2021). Furthermore, LAB maintain the production of valuable metabolites under diverse conditions by adapting their metabolic pathways to varying substrates and environmental pressures (acid- or bile-resistant salts). Metabolic flexibility and ability to grow of LAB in diverse environmental conditions make them ideal candidates for large-scale biotechnological applications. Zhou et al. (2021) discovered that combining NMN with *Limosilactobacillus fermentum* TKSN041 significantly reduced UVB-induced photoaging in mice by activating the AMPK (adenylate-activated protein kinase) signaling pathway, compared to using NMN or *L. fermentum* TKSN041 alone. This finding highlights the synergistic effect of LAB and NMN, indicating that employing LAB in NMN synthesis may enhance overall health benefits. Due to their promising characteristics of NMN production, research on NMN synthesis in LAB has emerged over the past 2 years, focusing mainly on strain selection and optimization of fermentation conditions (Sugiyama et al., 2021). Kong et al. (2023) utilized *L. lactis* NZ9000 as a starting strain, and optimized fermentation using the cost-effective substrate NAM, achieving an increase in NMN yields. With the in-depth research into the metabolic pathway of NMN, the biosynthesis approach for synthesizing NMN using LAB has emerged as the most promising large-scale production species for NMN. While promising, redirecting microbial metabolism to favor NMN production can place strain on cellular resources, potentially lowering growth rates and overall yield. Achieving a balance between cell health and product yield is essential for efficient production. Despite advancements, bioproduction yields often remain lower than chemically synthesized NMN, especially under industrial conditions. Metabolic engineering and pathway optimization remain critical areas for enhancing NMN output in microbial systems. Addressing these challenges is key to realizing the full potential of LAB as an NMN as a platform for NMN production. This review examines current solutions and future directions for optimizing LAB-based NMN biosynthesis.

## 2 Processes for NMN biosynthesis

NMN is naturally present in foods such as broccoli and shrimp, but the consumption of NMN from natural foods is insufficient to have a critical impact on human health (Table 1). Initially, NMN was synthesized through chemical synthesis, which posed several challenges, including the separation of chiral isomers, chemical residues, high production costs, and environmental concerns. In contrast, biosynthetic-utilizing natural products offer advantages such as enhanced safety, reduced raw materials costs, and minimal by-product formation (Yoshino et al., 2021). Although microbial synthesis may be initially more costly than chemical synthesis due to feedstock and process complexity, advancements in production efficiency and process optimization can eventually lead to reduced costs and improved economic viability. Additionally, the ability to convert certain by-products, such as adenosine monophosphate (AMP) and nicotinamide ribose, into valuable co-products promotes resource recycling and sustainability. Current biosynthesis preparation methods for NMN include two main methods: (1) Biocatalysis, which benefits from high specificity and mild reaction conditions but may face challenges related to enzyme stability and scalability (He et al., 2023; Qian et al., 2022). (2) Microbial fermentation, where engineered bacteria or LAB are employed to convert sugars or other precursors into NMN through metabolic engineering approaches (Kong et al., 2023; Shoji et al., 2021; Sugiyama et al., 2021).

**Table 1:** NMN present in various types of natural food.

Food name	mg/100 g—food	At least kg/300 mg supplementation
Edamame	0.47–1.88	16
Broccoli	0.25–1.22	26.8
Cucumber seed	0.56	53.6
Cucumber seed	0.65	46.2
Tomato	0.26–0.3	100
Avocado	0.36–1.60	18.8
Mushroom	0.0–1.01	29.7
Beef (raw)	0.06–0.42	71.4
Shrimp	0.22	136.4

### 2.1 Bioenzymatic synthesis of NMN

The biosynthetic pathway of NMN consists of multiple enzymatic catalytic steps, involving amidases, nucleotide phosphorylases, and phosphorylases. Depending on the core substrates that provide ribosyl and niacinamide, the current biocatalytic method is divided into two main types: one is based on the synthesis of NMN using PRPP and NAM as core substrates with the key enzyme NAMPT (Maharjan et al., 2021) and the other one is that NRK catalyzes the phosphorylation of NR with ATP to produce NMN (Cheng et al., 2024).

#### 2.1.1 Synthesis of NMN using NAM and PRPP as core substrates

**Figure 3:**
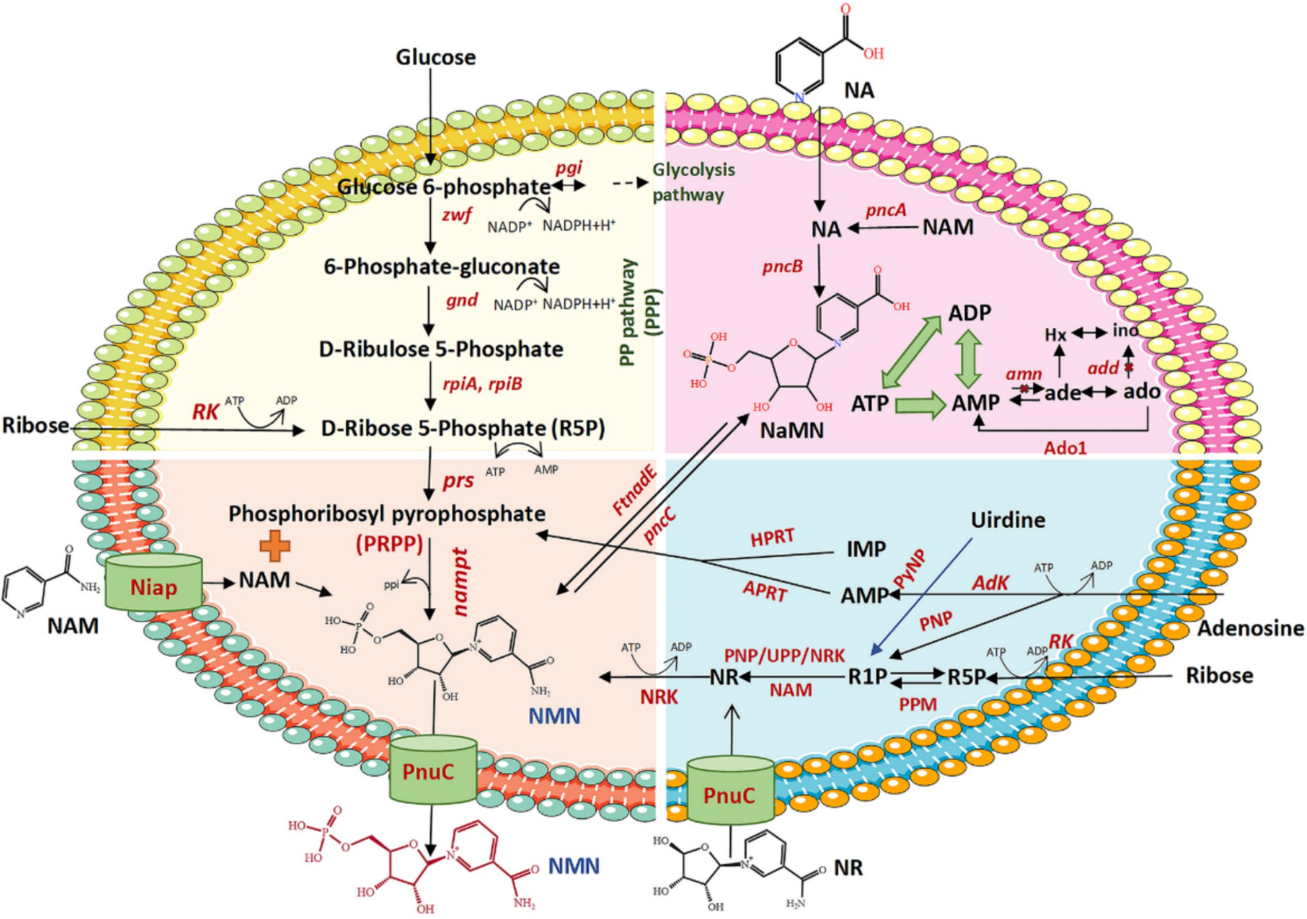
Schematic diagram of the biosynthesis of NMN using different substrates. *zwf*, glucose 6-phosphate dehydrogenase; *pgi*, glucose-6-phosphate isomerase; *pgl*, 6-phosphogluconolactonase; *gnd*, 6-phosphogluconate dehydrogenase; *rpiA*, ribose 5-phosphate isomerase A; *rpiB*, ribose 5-phosphate isomerase B; *prs*, phosphoribosyl pyrophosphate synthetase; NAMPT, nicotinamide phosphoribosyl transferase; R5P, ribose 5-phosphate; PRPP, 5-phosphoribosyl-1-pyrophosphate; NAM, nicotinamide; NMN, nicotinamide mononucleotide; PnuC, nicotinamide riboside transporter; NiaP, niacin transporter; *FtnadE*, NMN synthase from *Francisella tularensis*; *pncC*, NMN aminohydrolase; NA, nicotinic acid; NaMN, nicotinic acid mononucleotide; *pncA*, nicotinamidase; *pncB*, nicotinic acid phosphoribosyltransferase; NR, nicotinamide riboside; NRK, nicotinamide riboside kinase; RK, ribokinase; *amn*, AMP nucleosidase; *add*, adenosine deaminase; Ado1, adenosine kinase derived from *Saccharomyces cerevisiae*; R1P, ribose 1-phosphate; Adk, adenosine kinase; APRT, adenine phosphoribosyltransferase; ATP, adenosine triphosphate; ADP, adenosine diphosphate; AMP, adenosine monophosphate; PPi, inorganic pyrophosphate; IMP, inosine monophosphate; HPRT, hypoxanthine-guanine phosphoribosyltransferase; UPP, uridine phosphorylase; PyNP, pyrimidine nucleoside phosphorylase; PNP, purine nucleoside phosphorylase; PPM, phosphopentomutase.

Several biological pathways synthesize NMN via the intermediate PRPP, which can be converted to NMN by NAMPTs in the presence of NAM (Table 2 and Figure 3). Preiss and Handler (1957) reported that the synthesis of NMN is facilitated by NAMPT. As is well known, the NAMPT plays a crucial role in NAD metabolism and in maintaining cellular NAD levels. Huang et al. (2022) screened and evaluated eight NAMPTs from different sources, the nicotinamide phosphoribosyltransferase (NadV) from the Vibrio bacteriophage KVP40 (VpNadV) demonstrated notable activity in enhancing NMN accumulation in *E. coli*. Shoji et al. (2021) conducted a comparative analysis on the heterologous expression of 10 NAMPT enzymes from mammals and bacteria in *E. coli*, among which NAMPT from *Chitinophaga pinensis* was the best expression, with an enzyme activity 2.4 times greater than that of *Shewanella oneidensis*. Co-expression of the NadV with PRPP synthetase, combined with the addition of NAM, resulted in an NMN concentration of up to 4 mmol L^−1^ (International patent: US202016832347A) (Akiyama, 2020). In addition to direct screening, engineering wild-type NAMPT is considered essential for increasing its enzymatic activity (Huang et al., 2022). The catalytic activity of NAMPT from *Meiothermus ruber* DSM 1279 was increased by 1.2 to 6.9 times by the incorporation of mutations at key sites, including F180A, F180W, A182Y/E231A, E231Q, and D298A (Fu and Zhang, 2019). Furthermore, PRPP binds to the purine repressor *purR*, which regulates the transcription of the purine pathway, thereby consequently diminishing its affinity for NAMPT (Rappu et al., 2003; Zakataeva et al., 2012). Given the costly and unstable nature of PRPP as a direct substrate for NMN synthesis, there is increasing interest in utilizing more readily available and stable substrates such as ribose, adenosine, and glucose in this process.

1. Ribose as substrate (Figure 3, yellow and blue parts). An economic pathway for PRPP production is the use of ribose as a starting material to synthesize PRPP via two catalytic steps. First, the ribose being catalyzed by riboside kinase (RK) to produce D-ribose-5-phosphate (R5P) with the ADP as a by-product, using ATP as the phosphoryl donor (Tozzi et al., 2006). RK, which was widely found in *E. coli*, exhibits high catalytic activity in the presence of ATP, potassium, and magnesium ions (Chuvikovsky et al., 2006). Subsequently, R5P reacts with phosphoribosyl pyrophosphate synthetases (PRSs) to transfer the PPi moiety from ATP to the C1-hydroxyl group of R5P, ultimately yielding PRPP. RPSs are crucial enzymes responsible for PRPP generation (Jiang et al., 2017). PRSs are categorized into three groups, i.e., PRS1, PRS2, and PRS3. The PRS1s are among the best-studied and most widespread in bacteria and mammals. It can be activated by Mg^2+^ and phosphate but is competitively inhibited by ADP (de Brouwer et al., 2010; Jiang et al., 2017; Leone et al., 2015). In the case of PRS1, the diphosphorylation group is transferred exclusively from ATP to R5P, whereas PRS2 exhibits broader specificity for diphosphate donors (Chen et al., 2015). The NMN concentrations achieved only by insertion of the PRS2 gene (0.95 mmol L^−1^) in recombinant *E. coli* is comparatively higher than those resulting from expression of the PRS1 gene (0.82 mmol L^−1^) in the existence of both Mg^2+^ and phosphate. Moreover, co-expression of NAMPT with PRS1 and PRS2 led to a significantly higher NMN yield (2.31 mmol L^−1^) than the expression of either PRS1 or PRS2 alone, with the addition of ribose, Mg^2+^, and phosphate, and 0.5% NAM (Maharjan et al., 2021). Zakataeva et al. (2012) demonstrated that specific mutations in *Bacillus amyloliquefaciens* PRS (BaPRS), specifically N120S and L135I mutations (BaPRS^N120S^, BaPRS^L135I^), are conducive to the release the enzyme from ADP and GDP inhibition, remarkably increasing its sensitivity to activation of inorganic phosphate to promote synthesis of nucleosides compared with the wild-type BaPRS. Overexpression of the BaPRS^L135I^ in *E. coli* BL21 (DE3) increased NMN output to 22 mg L^−1^ with a 200 mg L^−1^ supplementation of NAM, which is approximately 6 times greater than the control (3.6 mg L^−1^) (Huang et al., 2022). These findings underscore the significant capacity of PRS enzymes to catalyze the conversion of ribose into PRPP through a cascade of reactions.
2. Adenosine as substrate (Figure 3, blue part). Adenosine is catalyzed to AMP by the enzyme adenosine kinase (AK), using ATP as a phosphate donor (Park and Gupta, 2008). The AK plays a key role in regulating the levels of adenosine and belongs to the same family of RK. Adenine and PRPP synthesis is catalyzed by AMP and PPi under the action of adenine phosphoribosyltransferase (APRT), facilitated by a divalent Mg^2+^ ion (Huyet et al., 2018). Within this process, the synthesis of AMP is considered a forward reaction, serving as a positive feedback inhibitor, with a higher concentration of AMP shown to enhance the synthesis of PRPP. Huyet et al. (2018) demonstrated that the Tyr105 of APRT is crucial for the fine-tuning of the catalytic efficiencies of the forward and backward reactions. The Tyr105Phe mutation has been found to amplify the inhibitory effect of AMP while reducing the catalytic efficiency of the enzyme by 11-fold in the forward reaction (Huyet et al., 2018). Ultimately, PRPP also generates NMNs under the role of NAMPT and NAM. A Chinese patent (publication number: CN202010058700.1) has described the utilization of adenosine as the core substrate, the final NMN concentration was optimized to be as high as 19.67 g L^−1^ based on the addition of the purification enzymes of AK, APRT, and NAMPT (2:1:1), with a purity of 54%, and a recovery rate of approximately 75–82%. With AMP as the core substrate and adding APRT and NAMPT mutant purified enzyme, the highest yield of NMN reached 32.3 g L^−1^ with a molar ratio of NAM:PPi:AMP = 1:4:1. In addition to AMP, inosine monophosphate (IMP) can also be used as a core substrate to produce PRPP and the by-product hypoxanthine via hypoxanthine phosphoribosyltransferase (HGPRT). The NMN can be synthesized from IMP- and NAM-catalyzed HGPRT and NAMPT (international patent: US202117164010A) (Zhang, 2021). However, adenosine costs are generally more cost-effective and widely applied in production than AMP or IMP as starting materials.
3. Glucose as substrate (Figure 3, yellow part). Glucose is one of the most accessible and inexpensive sugars, making it an ideal material for the synthesis of PRPP. The glucose is metabolized to generate R5P via the pentose phosphate pathway (PPP), which involves the enzymes such as phosphoglucose isomerase (*pgi*), glucose-6-phosphate dehydrogenase (*zwf*), 6-phosphogluconolactonase (*pgl*), 6-phosphogluconate dehydrogenase (*gnd*), ribose-5-phosphate isomerase A (*rpiA*), and ribose-5-phosphate isomerase B (*rpiB*). The R5P is subsequently converted to PRPP with the assistance of PRS and further transformed into NMN by NAMPT (Shoji et al., 2021). To enhance PRPP biosynthesis, the endogenous *E. coli* genes (*pgi*, *zwf*, *pgl*, *gnd*, *rpiA*, *rpiB*, and *prs*) were selected for artificial assembly followed by overexpression. In strains expressing only NAMPT, NMN production reached 75 mg L^−1^ of NMN from NAM and glucose, whereas the control lacking NAMPT did not produce NMN (Shoji et al., 2021). Co-expression of NAMPT alongside key genes of PPP, resulted in a 2.5-fold increase in NMN production compared with the above strains. It demonstrated that the expression of key genes of PPP is a reasonable strategy to enhance NMN biosynthesis. Overexpression of *zwf* or *gnd*, two major enzymes of the PPP, could improve carbon flux flow (Sekar et al., 2017; Wu et al., 2020). The highest yield of NMN was 496.2 mg L^−1^ from glucose and NAM, under optimized conditions of 37°C, pH 6.0, and OD_600_ = 50 (Liu et al., 2021).

**Table 2:** Comparison of NAMPT kinetic parameters from different sources.

Source	Activity U mg^−1^	*K*_m_ μmol L^−1^	*k*_cat_/*K*_m_ mmol L^−1^ s^−1^	References
*Chitinophaga pinensis* (WT)	0.89	5,220	0.61	Peng et al. (2024a)
*Chitinophaga pinensis* (Y15S)	1.4	4,920	0.64	Peng et al. (2024a)
Vibrio bacteriophage KVP40	0.82	NA	NA	Peng et al. (2024b)
*Haemophilus ducreyi* 35000HP	0.49	NA	NA	Peng et al. (2024b)
*Methanobacterium* sp. PtaU1.Bin097	0.37	NA	NA	Peng et al. (2024b)
*Mus musculus*	NA	0.92	2.17 × 10^7^	Revollo et al. (2004)
*Homo sapiens*	NA	0.0050	1.80 × 10^9^	Burgos and Schramm (2008)

NA, not available.

#### 2.1.2 Synthesis of NMN using NR as core substrates

Optimizing metabolic networks using multiple core substrates enhancing the precursor supply of PRPP, coupled with catalyzing reactions with NR substrates, could avoid the use of expensive and limited sources of PRPP (Huang et al., 2023). The conversion of NR to NMN via the catalysis of NRK in the presence of ATP is one of the most promising synthetic pathways for NMN production (He et al., 2023). The NRK belongs to the deoxynucleoside kinase and nucleoside monophosphate kinase superfamily and has been identified in various eukaryotes from yeast to humans (Table 3) (Eriksson et al., 2002). In humans, there are two forms: NRK1 and NRK2, both of which have highly conserved amino acid sequences (Bieganowski and Brenner, 2004; Khan et al., 2007). NRK1 is rate-limiting for utilizing exogenous NRs in the synthesis of NMN (Ratajczak et al., 2016). To address this limitation, He et al. (2022) engineered a whole-cell biocatalyst by exhibiting human NRK2 on the surface of *S. cerevisiae* EBY100, which whole-cell NRK-2 could powerfully convert NR to NMN, accompanied by a maximum activity of 64 IU g^−1^ and a *K*_m_ of 3.5 μmol L^−1^. Additionally, the NRK1 from *S. cerevisiae* was expressed in *E. coli*, and the specific enzyme activity of ScNRK1 after purification was 2252.59 IU mg^−1^ (He et al., 2023). Qian et al. (2022) identified a novel Klm-NRK from *Kluyveromyces marxianus* via two bioinformatic approaches (NCBI database and UniProt database), and the specific activity of the purified Klm-NRK was measured at 7.9 U mg^−1^ protein, ranking the highest among the reported NRKs. The enzymatic production of NMN was successfully achieved using 100 g L^−1^ NR as a substrate by adopting a novel and highly active NRK, resulting in a spatiotemporal titer of 281 g L^−1^ day^−1^ (Qian et al., 2022). It is demonstrated that the NRK holds great potential for the large-scale manufacturing of NMN. However, the predominant source of commercially available NR is chemical synthesis, which poses potential risks of chemical residues and comes with a higher price. Recent research has shown that the intermediate NR metabolite could be synthesized using ribose and riboside as substrates.

1. Ribose as substrate (Figure 3, blue part). The synthesis of NMN from ribose follows a pathway similar to that of PRPP production. Ribose uses ATP as the phosphate group donor to generate R5P and ADP as a by-product under the catalysis of RK; R5P is then converted to ribose-1-phosphate (R1P) under the action of phosphopentomutase (PPM); finally, the R1P synthesized NR under the action of dephosphorylation by NRK. A Chinese patent (Zhao Q. et al., 2021) (CN202110120111.6) demonstrated an efficient method for synthesizing NMN by employing RK, PPM, and NRK sequentially to convert ribose to NR and subsequently to NMN. This strategy bypasses the need to directly use the expensive NR as a raw material, obtaining 30.9 g of NMN with a reaction yield of 92.51%.
2. Nucleosides as substrate (Figure 3, blue part). Nucleosides and phosphates are stripped of their corresponding purines or pyrimidines to produce R1P by purine nucleoside phosphorylase (PNP) or pyrimidine nucleoside phosphorylase (PyNP) (Zhou et al., 2022); Then, the R1P is catalyzed by uridine phosphorylase (UPP) or PNP to produce the key intermediate NR with NAM and finally NR by the catalysis of NRK to synthesize NMN (Zhou et al., 2022). Zhou et al. (2022) designed a pathway for the NR phosphorylation to generate NMN, which can enhance the precursor supply of NR and achieve NMN production (3 g L^−1^) by a four-enzyme cascade catalytic system of PyNP, PNP, NRK, and polyphosphate kinase 2 after screening and system optimization.

**Table 3:** Comparison of NRK from different sources.

Sources	Activity (U mg^−1^)	*K*_m, ATP_ (μmol L^−1^)	*K*_m, NR_ (μmol L^−1^)	References
*Saccharomyces cerevisiae* NRK1	0.535	NA	NA	Bieganowski and Brenner (2004)
*Saccharomyces cerevisiae* (reScNRK1)	2252.59	0.05427	0.01869	He et al. (2023)
*Kluyveromyces marxianus* NRK	7.9	70	45	Qian et al. (2022)
Human NRK1	0.275	4.8	3.4	Dölle and Ziegler (2009)
Human NRK2	2.32	250	46	Dölle and Ziegler (2009)

NA, not available.

In summary, employing core substrates such as glucose, xylose, ribose, and nucleosides, in conjunction with NAM as co-substrate, enhances the supply of precursors for PRPP or NR (Table 4). This strategy enables enzymes to efficiently catalyze the synthesis of NMN.

**Table 4:** Price comparison of substrates and products.

Substrates/products	Source of the price	Price
Glucose	https://www.alibaba.com/product-detail/Glucose-Powder_11000013016284.html?spm=a2700.galleryofferlist.normal_offer.d_title.51662ef8KOfY8E	$0.57/kilogram
Xylose	https://www.alibaba.com/product-detail/Bulk-Price-Xylose-High-Quality-Assured_1600868801451.html?spm=a2700.galleryofferlist.normal_offer.d_title.5cda13a0EsNRUB	$1/kilogram
Nicotinamide (NAM)	https://www.alibaba.com/product-detail/Cosmetics-Grade-Vitamin-B3-Cosmetic-Ingredient_1601254466968.html?spm=a2700.galleryofferlist.p_offer.d_title.2ecd13a0S5CVfX&s=p	$12/kilogram
Adenosine triphosphate (ATP)	https://www.alibaba.com/product-detail/Factory-Supply-hot-selling-supply-Adenosine_1600529243610.html?spm=a2700.galleryofferlist.normal_offer.d_title.487c1eaf5qzHj4	$130/kilogram
Ribose	https://www.alibaba.com/product-detail/Ribose-D-ribose-Nutritional-Supplement-Sweetener_1600910476307.html?spm=a2700.galleryofferlist.p_offer.d_price.3fdc592bH4iN3H&s=p	$25/kilogram
adenosine	https://www.alibaba.com/product-detail/Adenosine-Best-Quality-Manufacturer-Adenosine-CAS_1601030100226.html?spm=a2700.galleryofferlist.p_offer.d_title.55216833QoAZ3g&s=p	$10/kilogram
NMN	https://www.alibaba.com/product-detail/Nmn-Best-Price-China-Factory-Nicotinamide_1600827100757.html?spm=a2700.galleryofferlist.p_offer.d_price.710e2021Kd1Y7V&s=p	$320/kilogram

### 2.2 Microbial fermentation

The biosynthesis of NMN using the microbial method has emerged as a hot spot of research due to its straightforward production process (Table 5). A critical factor for efficient NMN production is selecting appropriate chassis cells. Whole-cell biocatalysis, which utilizes intact microbial cells as biocatalysts, offers distinct advantages. These cells internally maintain the enzymes needed for NMN biosynthesis, simplifying enzyme immobilization and enabling continuous regeneration of cofactors, which reduce the reliance on costly additives. Engineered microorganisms, such as LAB or *E. coli*, can convert simple substrates into NMN through metabolic pathways. Zhao L. Q. et al. (2021) selected *Enterobacter chengduensis* 2021 T4.7 as a promising candidate, achieving NMN production through fermentation (67 μmol L^−1^) using NAM as a substrate. Several studies have focused on optimizing and intensifying the fermentative processes to enhance cell density and NMN yield (Maharjan et al., 2023; Kafle et al., 2023). For instance, Maharjan et al. (2023) reported a significant accumulation of NMN (51.6 mmol L^−1^) by implementing a linear feeding fed-batch strategy, resulting in a notable (22.4-fold) enhancement in NMN yield compared to flask-level experiments. To better understand the relationship between cell density and NMN production, the high cell density culture of *E. coil* was performed in a bioreactor with continuous feeding of NAM and glucose as co-substrates.

**Table 5:** Comparison of NMN biosynthesis cost using different approaches.

Substrate	Huang et al. (2022)	Tan et al. (2024)	Kong et al. (2024)	Su et al. (2024)
NAM (g/L)	8.3	—	1	5
Glucose (g/L)	27	45	20	—
Xylose	—	—	—	5
Yeast extract (g/L)	10	24	5	15
Tryptone (g/L)	—	12	10	—
IPTG (g/L)	0.119	—	—	—
Others (g/L)	29.658	14.84	20.84	10
NMN yield (g/L)	16.2	3.39	0.0534	0.4975
Cost ($/g NMN)	0.01644	0.05235	3.121	0.347

LAB strains, particularly *Lactobacillus* and *Bifidobacterium*, are increasingly recognized for their potential in NMN production and are classified as generally recognized as safe (GRAS) by regulatory bodies. This makes them suitable for applications in food and nutraceutical industries without major safety concerns. Known for their probiotic effects, these strains can contribute to improved gut health, immune modulation, and overall wellness. Incorporating NMN-producing LAB in functional foods could offer dual benefits: enhanced NAD metabolism and probiotic health effects. LAB can utilize inexpensive carbon sources such as lactose, glucose, or waste streams from food processing industries, making them a cost-effective option for NMN biosynthesis. The advantages of microbial fermentation include the use of renewable feedstocks (like glucose), reduced environmental impact due to mild operating conditions, and the potential for scalability with low-cost substrates. For example, Sugiyama et al. (2021) screened three NR-producing strains (genus *Fructobacillus*) from a library of 174 strains of LAB, using NR auxotrophic yeast as a screening tool, achieving a maximum NMN production of 2.1 mg L^−1^ NMN. However, achieving high NMN yields of LAB is often constrained by feedback inhibition, metabolic bottlenecks, and limitations in precursor availability. It requires fine-tuning metabolic pathways to enhance the flux toward NMN production without hindering cell growth.

## 3 Current strategies for enhancing NMN biosynthesis in LAB

### 3.1 Improved metabolic capacity of LAB

LAB have been extensively utilized in the food industry serving as starter cultures or probiotics for food fermentation (Gaspar et al., 2013; Mazzoli et al., 2014). The reconstruction of metabolic fluxes of key enzymes is anticipated to enhance the biosynthesis of NMN in LAB, potentially improving the probiotic and pharmacological effects of fermented foods. The NMN production in LAB is deeply connected to their central carbon metabolism as many of the key intermediates and energy sources needed for NMN biosynthesis are derived from central metabolic pathways. In LAB, glucose is metabolized through glycolysis, and a portion of glucose-6-phosphate is diverted into the PPP. Glucose-6-phosphate dehydrogenase initiates the PPP, producing ribulose-5-phosphate, which is then converted to R5P—a key precursor for NMN biosynthesis. Due to their preference for anaerobic or microaerophilic conditions, LAB have limited capacity for energy generation through oxidative phosphorylation, necessitating careful balancing of metabolic fluxes to maintain redox equilibrium. While the PPP continues to produce R5P in LAB, the absence of oxygen shifts its role primarily to NADPH production for biosynthesis rather than ATP generation. The constrained PPP flux under anaerobic conditions limits R5P availability, potentially impacting NMN biosynthesis. Engineering LAB to express enzymes such as transketolase and transaldolase could improve PPP flux, thus increasing R5P availability without causing an additional energy burden. Certain amino acids can contribute to NMN synthesis through their conversion to NaMN. LAB can utilize amino acids as carbon sources, linking protein metabolism to NMN production. Wang P. et al. (2024) reported that overexpression of quinolinate synthase (*nadA*); L-aspartate oxidase (*nadB*) and nicotinatenucleotide pyrophosphorylase (*nadC*) could enhance the accumulation of NMN in *E. coli* when supplemented with 1.00 g L^−1^ aspartic acid (Asp) substrate. Furthermore, to enhance NMN production in LAB, the carbon flux through central metabolic pathways can be redirected. Overexpression of *pncB* in *B. subtilis* WB600 can ensure more NA convert to NaMN to target NMN production (Tan et al., 2024). By knocking out or downregulating pathways that compete for precursors such as R5P or PRPP, the LAB can be engineered to maximize NMN production efficiency. Zhang S, et al. (2024) increased the yield of NMN by removing deaminase to change the synthetic flux of NAD. It indicated that limiting NAD consumption pathways can direct more resources toward NMN accumulation.

Gene overexpression strategies can effectively enhance the expression of key enzymes in the NMN biosynthetic pathway to improve NMN production. The key enzymes involved in NMN biosynthesis can be subjected to random mutagenesis to evolve variants with higher catalytic efficiency. In LAB, various constitutive promoters have been identified, such as P_11_ in *S. thermophilus*, which are capable of driving high levels of *epsA* and *epsE* gene expression to promote EPS synthesis (Kong et al., 2019). A Chinese patent (publication number: CN118460447B) has described that overexpression of the niacin transporter (Niap) lp2514 driven by the high-copy promoter P_23_ in *L. plantarum* WCFS1, and the final NMN production achieved was 160 μmol L^−1^. Furthermore, the recent development of CRISPR/Cas9-mediated genome editing systems for LAB has positioned them as highly promising microbial cell factories for future biorefineries (Hidalgo-Cantabrana et al., 2019; Huang et al., 2019; Oh and van Pijkeren, 2014; Song et al., 2021; Zhou et al., 2019). This advancement has expedited the precise genetic engineering of LAB, thereby facilitating comprehensive studies of their metabolic pathways and enabling the design of LAB cell factories for the production of NMN. Capozzi et al. (2011) successfully isolated *L. plantarum* capable of producing riboflavin, which increased the content of riboflavin in bread by approximately 3-fold. CRISPR/Cas9 specializes in precise genome editing combined with promoter engineering or plasmid-based systems to achieve maximum NMN yield in LAB. Consequently, these strategies can help balance the metabolic network, ensuring that sufficient precursors are directed toward NMN biosynthesis.

### 3.2 Future enhancement of NMN biosynthesis in LAB through metabolic engineering

Biosynthesis represents a relatively economical and environmentally sustainable approach, for synthesizing high value-added chemicals, with LAB emerging as a prominent contributor in this field (Kong et al., 2022b). To effectively produce NMN, it is crucial to regulate the metabolic network of the predominant microbial strain, thereby improving the conversion efficiency of the target products. Strategic application of genetic and metabolic engineering techniques can help achieve this goal. In terms of NMN biosynthesis, any metabolic modifications that promote the synthesis of the precursor PRPP or select key enzyme (NAMPT, FtNadE, NadR, etc.) will contribute to an increase in NMN production (Black et al., 2020; Stetsenko et al., 2020; Sugiyama et al., 2021). Engineering strategies could be applied to modify the NAD biosynthetic pathway to accumulate NMN, rather than converting it to NAD. Downregulation of specific genes, such as *nadR*, which are involved in NAD synthesis, or upregulating nucleoside triphosphate diphosphatase (MazG) could increase the availability of NMN. Kong et al. (2023) successfully enhanced NMN accumulation by 61% in *L. lactis* NZ9000 by knocking out *nadR* using the CRISPR/Cas9 system. Wang P. et al. (2024) found that overexpression of *mazG* diverted more flux to NMN and increased the NMN content two times. The NMN biosynthetic pathway of the candidate LAB RD012353 was elucidated, revealing the presence of seven proteins associated with “nicotinate and nicotinamide metabolism.” In particular, the identification of the key enzyme NAMPT, responsible for the production of NMN, which is commonly present in mammals and less reported in microorganisms (Table 6) (Sugiyama et al., 2021). To enhance the titer of NMN, 10 different NAMPT enzymes were screened and expressed in *E. coli* BL21(DE3). The NMN yields up to 75 mg L^−1^ by overexpressing the NAMPT-CP gene in BL21(DE3), while the control BL21(DE3) did not (Shoji et al., 2021). The NAMPT gene from *H. ducreyi* was overexpressed in *E. coli* BL21(DE3)pLysS, resulting in a remarkable 7.44-fold increase in NMN concentration compared to the control (Marinescu et al., 2018). Wang C. et al. (2024) found that the site-directed mutant CP-NAMPT D83N increased the NMN content by 3.7-fold (413.4 mg L^−1^) in *S. cerevisiae*. The synthesis of NMN also requires the participation of PRPP, which is the direct precursor of NMN. The PRSs were one of the most important enzymes required for PRPP generation (Weng et al., 1995). In strain NMN01, which only had NMAPT inserted extracellularly, NMN production reached 30.7 mg L^−1^. In contrast, NMN01 expressing PRS produced 79.8 mg L^−1^ (2.6-fold increase) (Liu et al., 2021). Increasing the pool size of PRPP could be achieved glucose fermentation. Overexpressing PPP artificial operons combined with CP-NAMPT resulted in a 2.5-fold greater in NMN production (189 mg L^−1^). Maharjan et al. (2021) observed a significant increase in NMN production (2.31 mmol L^−1^) when both NAMPT and PRS were co-expressed in recombinant *E. coli* cells. The PRS from *B. amyloliquefaciens* with L135I mutation and NAMPT gene from *H. ducreyi* were transformed in *E. coli* BL21(DE3)pLysS bringing the NMN yield of 17.26 mg per gram of protein to the highest level in 2018 (Marinescu et al., 2018). Huang et al. (2022) identified a *Vp*NadV gene that increased NMN yield, which was further improved to 22.2 mg L^−1^ when co-expressed with the BaPRS^L135I^. Through overexpression of CP-NAMPT D83N as well as enhanced PRPP supply, the intracellular NMN concentration was increased to 1.2 g L^−1^, which is currently the highest titer of NMN synthesis from inexpensive substrate (glucose and NAM) in *S. cerevisiae* (Wang et al., 2024; Wang et al., 2024). This underscores the promising potential of metabolic regulation in LAB to promote NMN synthesis.

**Table 6:** Effects of different metabolic engineering strategies and chassis on improving the production of NMN.

Substrates	Strategies	Enzyme source	Pathways	Chassis	NMN production	References
Nicotinamide (NAM)	Knockout *of nadR*	*L. latis* NZ9000	Salvage pathway	*L. latis* NZ9000	~0.2 g L^−1^ mg^−1^ protein	Kong et al. (2023)
NAM	Overexpression of FtnadE*	*FtnadE* from *F. tularensis*	Salvage pathway	*L. latis* NZ9000Δ*nadR*	0.765 g L^−1^mg protein	Kong et al. (2023)
Glucose, fructose	Fermentation	—	—	*F. tropaeoli* RD011727	1.5× 10^−3^ g L^−1^	Sugiyama et al. (2021)
Glucose, fructose	Fermentation	—	—	*F. durionis* RD012353	~2 × 10^−3^ g L^−1^	Sugiyama et al. (2021)
NAM, lactose	Overexpression *of nampt* and PRS^135I^	*nampt* gene from *Haemophilus ducreyi* and PRS^135I^ from *Bacillus amyloliquefaciens* with L135I mutation	Salvage pathway	*E. coli* BL21(DE3) pLysS	1.726 10^−2^ g L^−1^ protein	Marinescu et al. (2018)
Nicotinic acid (NA)	Overexpression *of nadV*	*nadV gene from Ralstonia solanacearum*	Salvage pathway	*E. coli*Δ*pncC*	—	Black et al. (2020)
NA	Overexpression *of nadV* and *FtnadE*	*nadV gene from R. solanacearum* and *FtnadE from F. tularensis*	Salvage pathway	*E. coli*Δ*pncC*	~0.5 g L^−1^	Black et al. (2020)
NAM, glucose	Overexpression *of nampt*, PRS^135I^, *zwf*, *gnd*, and YgcS; deletion of *pncC*, *yeeP*, *yjiV*, *yeeL*, and *nadR*	*nampt* gene from *H. ducreyi*; PRS^135I^ from *B. amyloliquefaciens* with L135I mutation; *zwf*, *gnd*, and YgcS from *E. coli* MG1655	Salvage pathway	*E. coli* MG1655 (NMN07-YgcS)	~0.3 g L^−1^	Liu et al. (2021)
NAM, glucose	Overexpression *of ado1*; deletion of *amn* with optimized condition	*ado1* gene from *Saccharomyces cerevisiae*	Salvage pathway	*E. coli* NMN07Δamn: P_119_-*ado1*	0.4962 g L^−1^	Liu et al. (2021)
Ribose, NAM	Overexpression *of nampt-prs1-prs2*	*nampt*, *prs1* and *prs2 from Homo sapiens*	Salvage pathway	*E. coli* BL21(DE3)	0.772 g L^−1^	Maharjan et al. (2021)
NAM, glucose	Overexpression of *nampt*, *niaP*, *pnuC*, *pgi*, *zwf*, *pgl*, *gnd*, *rpiA*, *rpiB*, and *prs*	*nampt* gene from *Chitinophaga pinensis*; *niaP* from *Burkholderia cenocepacia*; *pnuC* from *Bacillus mycoides*	Salvage pathway	*E. coli* BL21(DE3)	6.79 g L^−1^	Shoji et al. (2021)
NAM	Overexpression of *VpnadV*, BaPRS^L135I^, and BMpnuC in a shake flask	Vp*nadV* gene from *Vibrio bacteriophage* KVP40; PRS^135I^ from *B. amyloliquefaciens* with L135I mutation; BMpnuC from *B. mycoides*	Salvage pathway	*E. coli* BL21(DE3)	2.6 g L^−1^	Huang et al. (2022)
NAM	Overexpression of VpNadV, BaPRS^L135I^, and BMpnuC in a 5-L bioreactor	VpnadV from *V. bacteriophage* KVP40; PRS^135I^ from *B. amyloliquefaciens* with L135I mutation; BMpnuC from *B. mycoides*	Salvage pathway	*E. coli* BL21(DE3)	16.2 g L^−1^	Huang et al. (2022)
None	Overexpression of *nampt* and BMpnuC	*nampt gene from C. pinensis*; *pnuC from B. mycoides*	Salvage pathway	*E. coli* BL21(DE3)	0.24 g L^−1^	Bi et al. (2023)
NAM, glucose	Overexpression of *nampt*, BMPnuC, *zwf*, *gnd*, *prs*, and *ado1*; Knockout of *amn* and *add*	*nampt gene from C. pinensis*; BMpnuC *from B. mycoides*; *ado1* gene from *S. cerevisiae*; *zwf*, *gnd*, and *prs* gene *from E. coli* BL21(DE3)	Salvage pathway	*E. coli* BL21(DE3)	1.1 g L^−1^	Bi et al. (2023)
D-xylose, NAM	Overexpression of *xylA*, *xylB*, *rpe*, *rpiA*, *rpiB*, *prs*, and *nampt*; Knockout of *hisG*, *hpt*, *apt*, *tktB/talA*, *talB*, *pncC*, and *nadR* in the optimized system	*nampt gene from C. pinensis*; *xylA*, *xylB*, *rpe*, *rpiA*, *rpiB*, and *prs from E. coli* BL21(DE3)	Salvage pathway	*E. coli* BL21(DE3)	0.4975 g L^−1^	Su et al. (2024)
D-xylose, NAM	Overexpression of *xylA*, *xylB*, *rpe*, *rpiA*, *rpiB*, *prs*, and *nampt*; Knockout of *hisG*, *hpt*, *apt*, *tktB/talA*, *talB*, *pncC*, and *nadR* in the 5 L-bioreactor	*nampt gene from C. pinensis*; *xylA*, *xylB*, *rpe*, *rpiA*, *rpiB*, and *prs from E. coli* BL21(DE3)	Salvage pathway	*E. coli* BL21(DE3)	0.7602 g L^−1^	Su et al. (2024)
Glucose	Overexpression of *pncB*, *nadE*, and *pnuC*	*pncB and nadE from Bacillus subtilis*; *pnuC from B. mycoides*	*de novo* pathway	*B. subtilis* WB600	3.396 g L^−1^	Tan et al. (2024)
Nicotinamide ribose, ATP	Overexpression of NRK	NRK from *Homo sapiens*	Salvage pathway	*S. cerevisiae* EBY100	12.6 g L^−1^	He et al. (2022)
Aspartic acid	Overexpression of *nadABC*, *nadDE*, and MazG	*nadABC*, *nadDE*, and MazG gene from *E. coli* ATCC8739	*de novo* pathway	*E. coli* 8,739(DE3)	0.153 g L^−1^	Wang P. et al. (2024)
Asp, NAM, glucose	Overexpression of *nadABC*, *nadDE*, MazG, *nadV*, *niaP*, *pnuC*, *zwf*, *gnd*, and *prs*	*nadABC*, *nadDE zwf*, *gnd*, and MazG gene from *E. coli* ATCC8739; *nadV* from *C. pinensis*, *niaP* from *B. cenocepacia*, and *pnuC* from *B. mycoides*; *prs* from *P. calidifontis*	Salvage pathway and *de novo* pathway	*E. coli* 8,739(DE3)	0.781 g L^−1^	Wang P. et al. (2024)

ND, not determined; tktB, transketolase 2; talA, transaldolase A; talB, transaldolase B; amn, AMP nucleosidase; add, adenosine deaminase; ado1, adenosine kinase; xylA, xylose isomerase; xylB, xylulo-kinase; rpe, ribulose 3-phosphate isomerase; hisG, ATP phosphoribosylase; apt, adenine phosphoribosyl transferase; hpt, hypoxanthine-guanine phosphoribosyl transferase.

The NMN synthase from *Francisella tularensis* (*Ft*NadE*) has demonstrated the ability to convert nicotinic acid mononucleotide (NaMN) directly into NMN without NAD synthesis both *in vitro* and *in vivo* (Sorci et al., 2009). It is the most promising route for the *de novo* synthesis of NMN is the amidation of NaMN catalyzed by *Ft*NadE*(Sorci et al., 2009). Expression of *Ft*NadE* with codon optimization was performed in *L. lactis* NZ9000Δ*nadR*, confirming its significant advantage in increasing NMN titer to 2,289 μmol L^−1^ mg^−1^ protein via optimization of fermentation conditions. Meanwhile, the key genes (*pncA*, *nadD*, and *prs1*) involved in the NMN biosynthesis pathway were upregulated. Co-expression of *R. solanacearum* NadV and *Ft*NadE* resulted in the NMN reaching approximately ~1.5 mmol L^−1^ in *E. coli*, representing a 130-fold enhancement over the basic NMN level of strains. The production of NMN reached 33.2 mg L^−1^ when the key enzyme *Ft*NadE and nicotinic acid phosphoribosyltransferase (*pncB*) were expressed in *E. coli* (Huang et al., 2022), compared to the control level of 3.6 mg L^−1^. In *B. subtilis*, expression of *pncB* was markedly increased the titer of NMN (Zhang S, et al., 2024). It is evident that the synthesis of NMN is affected by a range of genes in microorganisms, which can substantially enhance in NMN production by the regulation of metabolism (Figure 3 and Table 6) (Camarca et al., 2021; Peng et al., 2024b). Successful metabolic engineering in *E. coli*, *S. cerevisiae*, or *B. subtilis* may be relevant or adaptable to LAB, including strategies like enhancing NMN biosynthetic enzymes or redirecting carbon flux toward NMN precursors. However, the synthesized NMN is not efficiently transported outside the cell, which can hinder NMN biosynthesis effectiveness. Previous studies have shown that introducing efficient transporters can effectively increase product biosynthesis (Huang et al., 2022; Shoji et al., 2021). Shoji et al. (2021) reported that an NMN transporter from *Bacillus mycoides* (BMpnuC), which can effectively transport the intracellular NMN into the extracellular environment. When the BMpnuC was expressed in *E. coli*, the extracellular NMN accumulation increased substantially from 0.124 g L^−1^ to 1.7 g L^−1^, demonstrating that efficiency of BMpnuC in exporting NMN (Huang et al., 2022). The titer of NMN was further elevated to 16.2 g L^−1^ by optimizing the fed-batch process (Huang et al., 2022), representing the highest yield of NMN synthesized from NAM in *E. coli* reported to date (Table 6). Adapting similar strategies in LAB, such as modifying the membrane transport systems or incorporating synthetic NMN export pathways, could enable these strains to secrete NMN directly into the medium, thereby bypassing intracellular NAD conversion. This approach would allow LAB to reduce the need for cell lysis during downstream processing. Combining fermentation techniques such as batch feeding and continuous culture with metabolic engineering, LAB can be fine-tuned to produce higher NMN yields, positioning them as viable candidates for industrial-scale production.

### 3.3 Challenges associated with increasing NMN biosynthesis in LAB

**Figure 4:**
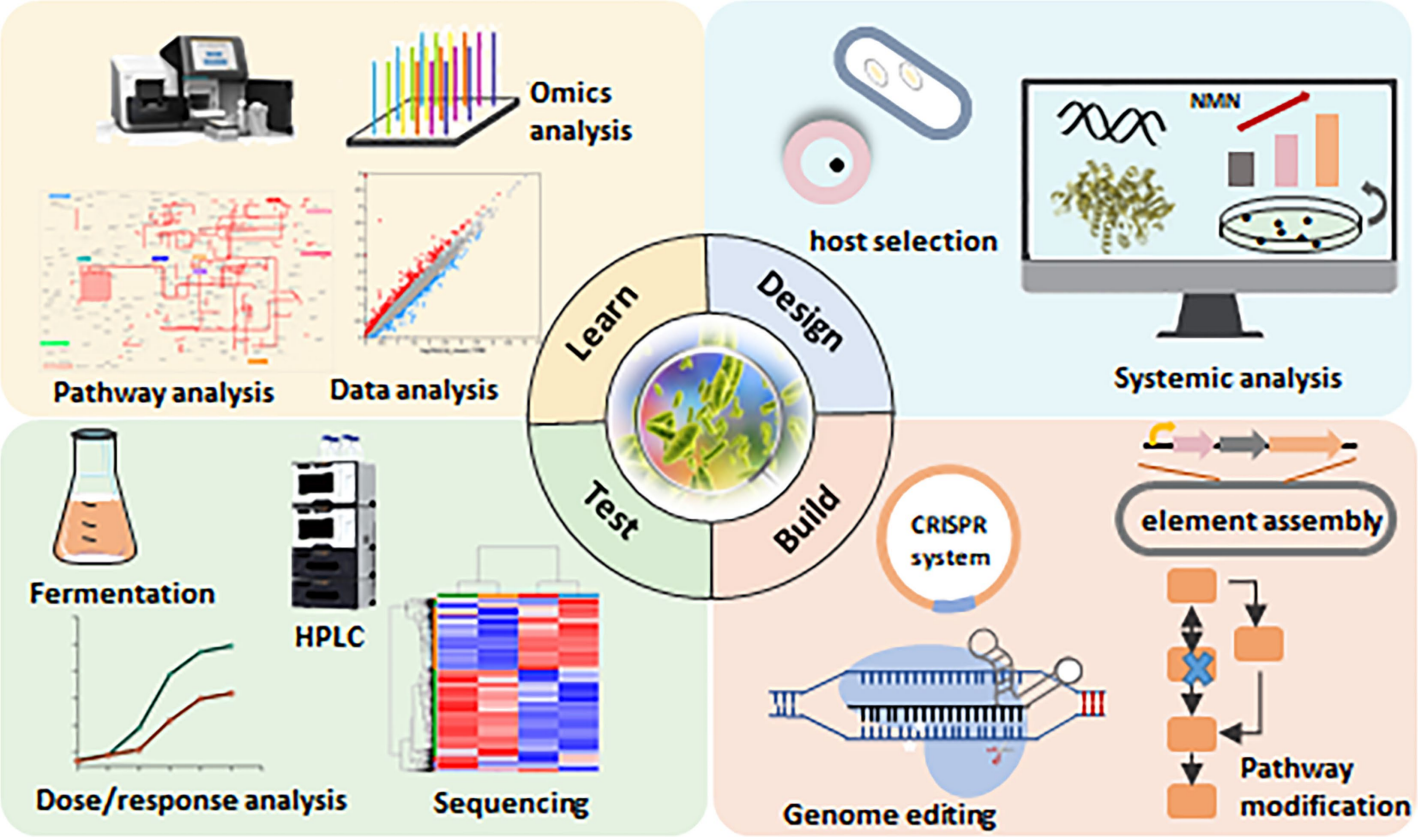
Synthetic biology approaches for NMN production in lactic acid bacteria (LAB). The design-build-test-learn (DBTL) framework is a well-established method in metabolic engineering. This diagram illustrates the DBTL cycle applied to NMN biosynthesis in LAB, highlighting key components of each stage. “Design”: selection of suitable hosts for NMN synthesis through systematic analysis. “Build”: modification of the NMN synthesis pathway using the CRISPR/Cas9 system. “Test”: verification of NMN synthesis capacity through fermentation. “Learn”: application of machine learning and statistical methods to identify the relationship between NMN levels and the design parameters.

Increasing NMN production in LAB through metabolic engineering presents several challenges related to the allocation of cellular resources, energy, and metabolic flux. Overproduction of NMN can disrupt the balance of cofactors such as NAD and ATP, which are required for various other cellular reactions. This imbalance may reduce the efficiency of central metabolic pathways, forcing the cells to work harder to maintain homeostasis and further increasing the metabolic burden. To address these challenges, it is crucial to redistribute metabolic flux, for example, by balancing the glycolytic pathway with the PPP, ensuring that NMN production does not draw off all available resources. Additionally, PRPP synthetase can also be inhibited by high levels of downstream products, including NAD or nucleotides. Integrating dynamic regulation systems and balancing precursor supply with cellular cofactor needs can help sustain high levels of NMN production without compromising cellular health or metabolic efficiency. Moreover, screening for LAB strains with inherently higher genetic stability is vital for the long-term success of engineered NMN production. The application of synthetic biology tools, such as real-time monitoring and process optimization, can ensure that engineered LAB strains remain robust, productive, and efficient over extended production cycles. Optimizing the fermentation conditions (pH, temperature, and nutrient availability) for LAB can significantly enhance NMN yields by directly affecting enzyme activity, cellular metabolism, and overall productivity. Maintaining an optimal pH can help minimize inhibitory by-products and reduce metabolic stress, enabling higher cell densities and potentially increasing NMN production. Most LAB strains exhibit optimal growth and metabolic activity between 30°C and 37°C, using stepwise temperature shifts during different fermentation stages based on strain-specific characteristics can enhance enzyme activity in NMN biosynthetic pathways, leading to higher yields. Optimizing glucose concentrations to prevent excess metabolic burden or carbon overflow or to supply NMN precursors such as NAM could contribute to the maintenance of growth and NMN biosynthesis. Continuous fermentation is particularly well-suited for large-scale operations, as the uninterrupted supply of fresh medium with optimal nutrient and precursor concentrations maintains the cells in their most efficient phase. This makes it a viable strategy for the commercial production of NMN. Furthermore, the integration of artificial intelligence (AI) and machine learning (ML) into metabolic engineering represents a transformative approach for researchers seeking to design and optimize microbial strains for enhanced production of NMN in LAB (Figure 4). By leveraging large-scale multi-omics data (genomics, transcriptomics, proteomics, and metabolomics) and predictive modeling, ML-based approaches can streamline the rational design of high-yield strains of NMN while reducing uncertainties. The ML-driven metabolite profiling systems can efficiently process high-throughput data from engineered strain libraries, rapidly identifying strains with the highest NMN yields. Combining systems biology with AI, it will not only enhance our understanding of LAB metabolism but also guide precise and efficient engineering strategies to unlock the full potential of LAB for NMN biosynthesis in industrial applications. This will provide a roadmap for future efforts to establish LAB as a viable microbial factory for large-scale, food-grade NMN production.

## 4 Conclusion and perspectives

Currently, there are still relatively lack of studies on NMN biosynthesis in LAB, with recent research principally focused on *E. coli*, which limits the application of NMN in the dairy industry. This review highlights the potential of metabolic engineering strategies to enhance NMN production in LAB, emphasizing the advantages, such as probiotic benefits and established safety, making them promising candidates for NMN production. However, challenges remain, including strain stability and metabolic burden. Accordingly, future research should concentrate on two main areas: (1) Developing innovative strategies, such as exploring alternative gene regulation techniques, engineering feedback-resistant enzymes, and utilizing systems biology approaches to balance growth and production. (2) Using AI-driven models to optimize metabolic pathways, predict strain behavior, and reduce experimental cost and uncertainty, thereby facilitating the development of a genetically stable probiotic cell factory. This review aimed to refine the fermentation process and facilitate the large-scale biosynthesis of NMN in LAB to achieve its industrial application in dairy products.
